# Angiosarcoma With Malignant Peripheral Nerve Sheath Tumour Developing in a Patient With Klippel–Trénaunay–Weber Syndrome

**DOI:** 10.1080/13577140500353743

**Published:** 2005

**Authors:** Marieke J. M. Ploegmakers, Maciej Pruszczynski, Jacky De Rooy, Benno Kusters, René P. H. Veth

**Affiliations:** 1 Department of Radiology Radboud University Medical Centre Nijmegen Post-box 9101 Nijmegen NL-6500 HB The Netherlands; 2 Department of Pathology Radboud University Medical Centre Nijmegen Post-box 9101 Nijmegen NL-6500 HB The Netherlands; 3 Department of Orthopaedics Radboud University Medical Centre Nijmegen Post-box 9101 Nijmegen NL-6500 HB The Netherlands

## Abstract

*Purpose:* We discuss the coexistence of Klippel–Trénaunay–Weber syndrome with various malignancies, the possible histogenetic pathways and therapeutic implications.

*Patient:* We report on a 46-year-old man presenting with increasing pain and swelling of his right lower leg after fracturing his fibula. Since birth he was known as having the uncommon syndrome of Klippel–Trénaunay–Weber of his right lower leg.

*Methods:* Our patient underwent an above-knee amputation for biopsy-proven malignant vascular tumour, first thought to be a composite hemangio-endothelioma and/or angiosarcoma with lung metastases.

*Results:* In the amputated extremity, a vascular malformation was found with tumour showing various components with foci of angiosarcoma adjacent to diffuse neurofibroma and areas with high-grade malignant peripheral nerve sheath tumour. Amputation and palliative chemotherapy were indicated, but he died of pulmonary and cerebral metastases 2 months postoperatively.

*Discussion:* This case describes an angiosarcoma with malignant peripheral nerve sheath tumour developing in a patient with Klippel–Trénaunay–Weber syndrome. A case never described before in literature and probably, as in our case, difficult to diagnose at first biopsy.


*Sarcoma*, September/December 2005; 9(3/4): 137–140

Taylor & Francis

Taylor & Francis Group

**ORIGINAL ARTICLE**

### Angiosarcoma with malignant peripheral nerve sheath tumour developing in a patient with Klippel–Trénaunay–Weber syndrome

MARIEKE J. M. PLOEGMAKERS[1], MACIEJ PRUSZCZYNSKI[2], JACKY DE ROOY[1], BENNO KUSTERS[2], & RENÉ P. H. VETH[3]

[1]*Department of Radiology,* [2]*Department of Pathology, and* [3]*Department of Orthopaedics, Radboud University Medical Centre Nijmegen, Post-box 9101, NL-6500 HB, The Netherlands*

*(Received 29 August 2005)*

**Abstract**

*Purpose*: We discuss the coexistence of Klippel–Trénaunay–Weber syndrome with various malignancies, the possible histogenetic pathways and therapeutic implications.

*Patient*: We report on a 46-year-old man presenting with increasing pain and swelling of his right lower leg after fracturing his fibula. Since birth he was known as having the uncommon syndrome of Klippel–Trénaunay–Weber of his right lower leg.

*Methods*: Our patient underwent an above-knee amputation for biopsy-proven malignant vascular tumour, first thought to be a composite hemangio-endothelioma and/or angiosarcoma with lung metastases.

*Results*: In the amputated extremity, a vascular malformation was found with tumour showing various components with foci of angiosarcoma adjacent to diffuse neurofibroma and areas with high-grade malignant peripheral nerve sheath tumour. Amputation and palliative chemotherapy were indicated, but he died of pulmonary and cerebral metastases 2 months postoperatively.

*Discussion*: This case describes an angiosarcoma with malignant peripheral nerve sheath tumour developing in a patient with Klippel–Trénaunay–Weber syndrome. A case never described before in literature and probably, as in our case, difficult to diagnose at first biopsy.

**Keywords:** *Angiosarcoma, malignant peripheral nerve sheath tumour, Klippel–Trénaunay–Weber*

### Introduction

An angiosarcoma with malignant peripheral nerve sheath tumour developing in a patient with Klippel–Trénaunay–Weber syndrome has never been described before in the literature. We discuss the coexistence of Klippel–Trénaunay–Weber syndrome with various malignancies, possible histogenetic pathways and therapeutic implications.

### Case

A 46-year-old man had been known since birth in various hospitals with a hypertrophy of the right lower leg and foot diagnosed as Klippel–Trénaunay–Weber syndrome (KTW). During childhood the lesion was suspected as an arteriovenous fistula, although angiography and oscillography did not reveal abnormalities. Eventually, hypertrophy necessitated epiphysial arrest operations of the right proximal tibia and, 3 years later, a distal epiphysial arrest of the right tibia and fibula because of hypertrophy. In addition he sustained a trauma resulting in an ankle joint deformity. During adolescence, the right leg again demonstrated abnormal growth in combination with oedema. At that time angiography showed an oscillating course of the anterior tibial artery. Excision of an arteriovenous malformation was performed. Several days after this procedure, the leg was thinner and the oedema had almost completely dissolved. A year after the operation, a painful swelling appeared spontaneously, distal to the lateral malleolus. Palpation showed a vast, mobile and bean-sized swelling in the soft tissues. The complaints were attributed to a tendovaginitis and treated conservatively.

Correspondence: Professor R. P. H. Veth, MD PhD, Department of Orthopaedics 800, Radboud University, Nijmegen Medical Centre, Post-box 9101, NL-6500 HB Nijmegen, The Netherlands. Tel: 31 24 3614480. Fax: 31 24 3540230. E-mail: r.veth@orthop.umcn.nl

ISSN 1357–714X print/ISSN 1369–1643 online/05/(03-04)0137-4 © 2005 Taylor & Francis
DOI: 10.1080/13577140500353743

Years later, a triple arthrodesis of the foot and lateral ligament plasty were performed because of laxity of the lateral ligaments and valgus position of the ankle during walking. At that time, the circumference of the right lower leg was equal to that of the left lower leg. At the age of 46, he suffered from progressive pain and swelling of his right lower leg. The working diagnosis was pseudo-Kaposi sarcoma in a patient with KTW syndrome. Biopsies of the soft tissues and bone were taken at a local hospital. Histology revealed an angiosarcoma. In the next few weeks, the leg became extremely swollen and the pain increased. He lost 20 kg of bodyweight in 4 months, felt weak and perspired.

On admission to our university hospital, physical examination showed an extensive swelling of the right lower leg, a blue skin, intact peripheral pulsations and a non-painful fixed firm-elastic tumour. Plain X-ray images revealed destruction of talocrural and subtalar joints, permeative cortical destruction with periosteal reaction of the tibia, and calcifications in a pretibial soft tissue swelling, suggesting malignancy or osteomyelitis. A technetium total body bone scan showed irregularities in the lower leg and hotspots in the tibia and fibula. MR imaging (Figure 1) revealed relatively well-defined soft tissue masses with heterogenic signal intensities and heterogenic contrast enhancement. Cortical erosion with intra-osseous bone marrow changes was obvious. Taking into account the case history the differential diagnosis included soft tissue malignancy, probably of vascular origin, with intra-osseous extent. A chest X-ray was suspect for metastases in both lungs. Reassessment of the biopsies taken at the local hospital revealed a vascular tumour with some spindle cell component thought to be a composite hemangio-endothelioma and/or angiosarcoma.

Because of severe pain, the oncology team and the patient decided to perform palliative treatment by means of above-knee amputation with postoperative chemotherapy.

### Histopathology

The amputated leg showed a fleshy, partly haemorrhagic, soft tissue lesion extending from the knee to the foot. Microscopically, a vascular malformation was seen, composed of dilated blood vessels with variably thickened walls, mixed with bland-looking diffuse neurofibroma with occasional Meissner corpuscles. In some areas there was a transition to fascicular high-grade spindle cell sarcoma with high mitotic activity and positive S100 protein confirming nerve sheath differentiation (Figure 2A). This tumour component also showed whorled structures, often with perivascular growth and neural-like morphology. Additionally, there were foci of a malignant tumour with vasoformative characteristics,

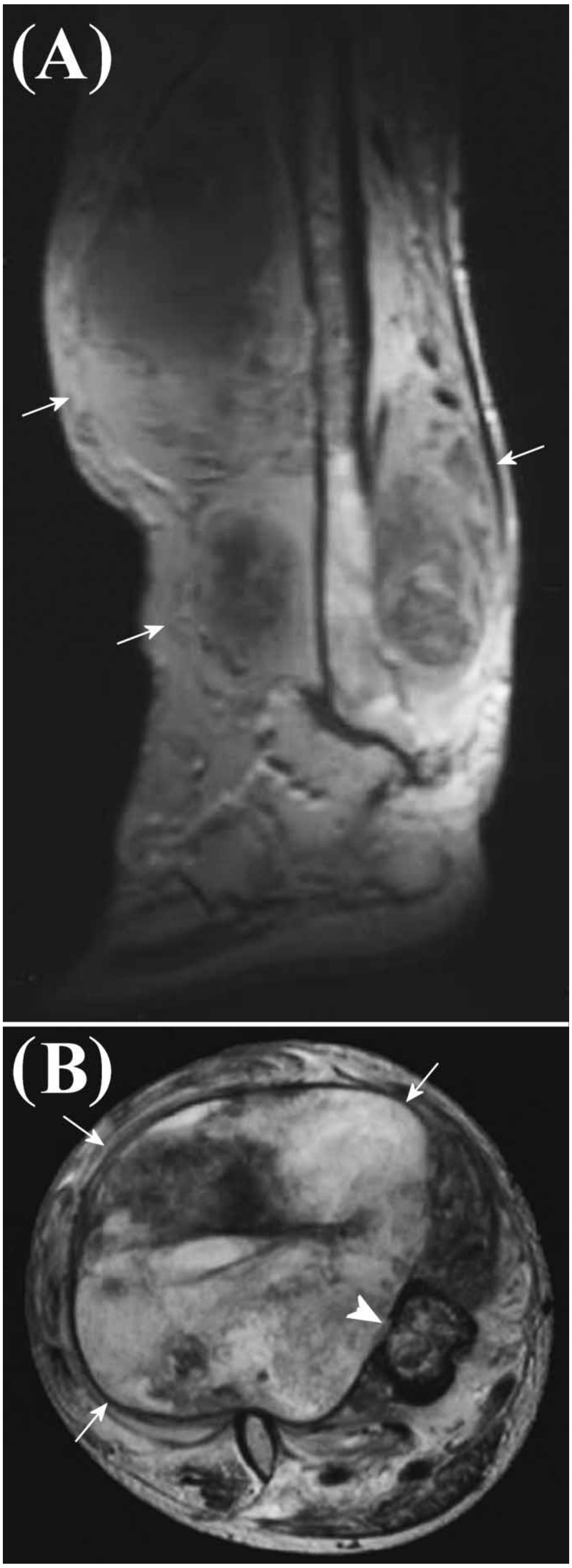

Figure 1. (A) Sagittal gadolineum enhanced T1-weighted image of the right lower leg shows relatively well delineated soft tissue masses (arrows) with diffuse contrast enhancement (high signal) as well as cortical erosion and bone marrow changes. (B) T2-weighted image of the right lower leg shows a large soft tissue mass (arrows) with heterogenic signal intensities causing clear erosion of the adjacent cortex (arrowhead) of the deformed tibia as well as bone marrow changes.

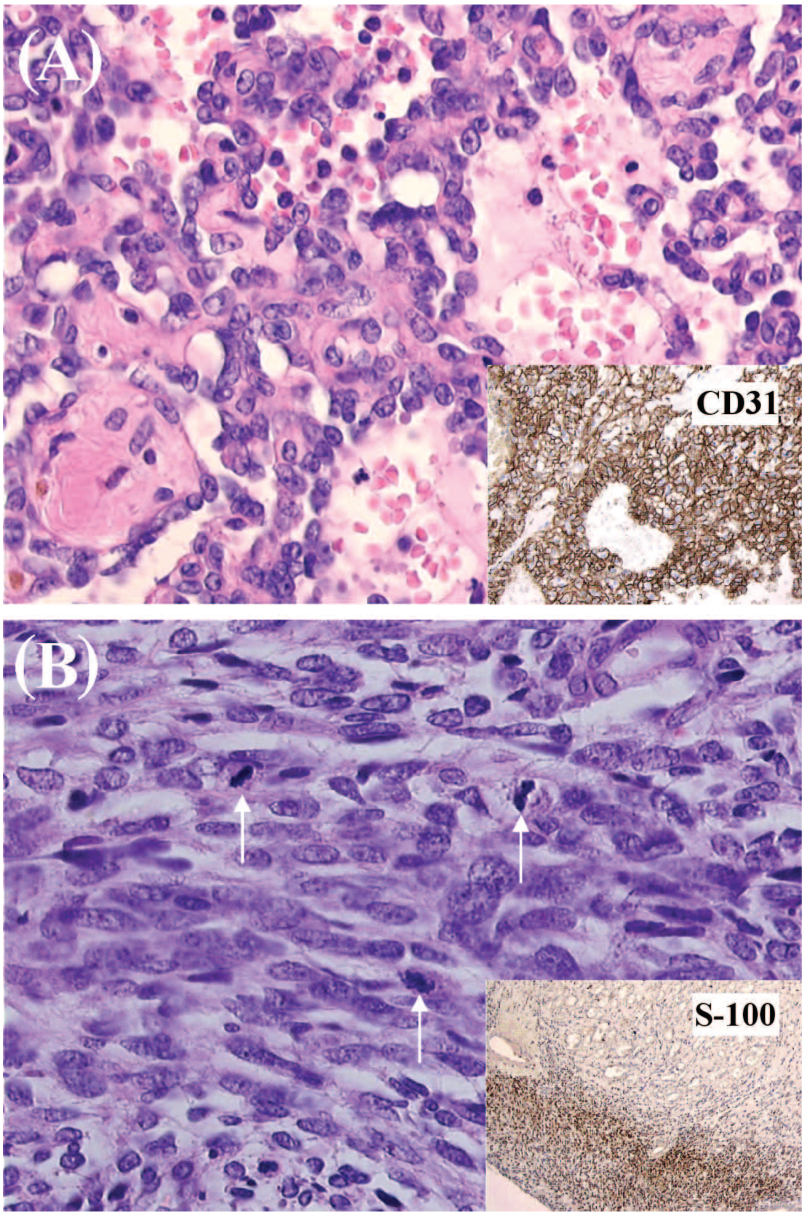

Figure 2. Histological pictures of the tumor (H&E and immunohistochemistry). (A) The vascular compartment with atypical endothelial cells (positive for CD31, inset). (B) The nerve sheath compartment of the tumor. The atypical spindle-shaped cells are frequently positive for S-100, as shown in the inset. Note that in the upper part of the inset in (B), the vascular compartment is negative for S-100. Arrows indicate mitotic figures.

lumina formation and intravascular papillary excrescences, showing convincing positive endothelial marker CD31 (Figure 2B). Both malignant components were highly immunoreactive for tumour proliferation marker MIB1. A soft tissue tumours expert confirmed our diagnosis of high-grade malignant peripheral nerve sheath tumour (MPNST) with angiosarcomatous differentiation developing in a vascular malformation with diffuse neurofibroma. Additionally, dermal haemangiomas and some reactive bone formation in the soft tissue were found, as well as extension of the lesion in the tibia. Three lymph nodes and surgical margins were free of tumour.

### Follow-up

One month after the operation the patient became dyspnoeic and suffered from intense headaches. Computed tomography (CT) of the thorax showed bilateral hydrothorax and extensive pulmonary metastases. Contrast-enhanced CT of the brain showed cerebral metastases. He died 5 weeks after the operation.

### Discussion

The KTW syndrome is a rare congenital condition of obscure aetiology and variable expression, most typically characterised by the triad of cutaneous haemangiomas, varicose veins, limb hemihypertrophy and often arteriovenous malformation.

In the literature there are casuistic reports on tumours associated with KTW syndrome, e.g., Hodgkin's lymphoma [1], rhabdomyosarcoma [2], squamous cell carcinoma of the skin [3], astrocytoma [4], meningioma [5], haemangiopericytoma [6] and angiosarcoma [7]. One patient has been described with Klippel–Trénaunay–Weber syndrome, who received previous radiation therapy to treat a benign haemangioma 1 year before the diagnosis of angiosarcoma [8]. Recently a case was reported on neurofibrosarcoma, which had developed in a setting of neurofibromatosis in a patient with KTW syndrome [9].

An angiosarcoma rarely develops in the setting of neurofibroma, neurofibromatosis type 1 or 2 or malignant peripheral nerve sheath tumour (MPNST) [10]. Our patient had diffuse neurofibroma, but not neurofibromatosis type 1 or 2. About one-third of soft tissue angiosarcomas develop in association with other neoplasms, genetic syndromes or synthetic vascular grafts [7].

In our case, we found the very unusual combination of MPNST with heterologous angiosarcomatous differentiation developed in a vascular malformation with diffuse neurofibroma. Whether this angiosarcoma developed in a pre-existent angiodysplastic lesion or as a result of divergent differentiation of neurogenic tumour is a matter of speculation. Malignant change in a pre-existent benign vascular tumour is probably an unusual event. Only about 15 convincing cases have been published until now [11]. The combined tumour in our patient was difficult to classify at first biopsy. Only in the amputation specimen, after examination of large series of slides, could the exact nature of the tumour be fully specified.

Radiological diagnosis included soft tissue malignancy probably of vascular origin. Radiological analysis did not contribute to the histopathological diagnosis, as it was only able to show the delineation of the soft tissue changes.

For both angiosarcomas and MPNST, the surgical treatment consists of a wide resection, followed by local radiation therapy and systemic chemotherapy.

### Acknowledgement

The authors would like to thank Professor Christofer D.M. Fletcher, M.D. FRCPath, of the Department of Pathology of Harvard Medical School in Boston for his highly appreciated expert consultative opinion and comments on the reported case.

### References

1. Aygencel G, Demiroglu H. Hodgkin disease in patient with Klippel–Trénaunay syndrome. N Z Med J 1997;110:133.
2. Fay A, Fynn-Thompson N, Ebb D. Klippel–Trénaunay syndrome and rhabdomyosarcoma in a 3-year-old. Arch Ophtalmol 2003;121:727–729.
3. De Simone C, Giampetruzzi AR, Gueriero C, De Masi M, Amerio P, Cina G. Squamous cell carcinoma in a venous ulcer as a complication of the Klippel–Trénaunay syndrome. Clin Exp Dermatol 2002;27:209–211.
4. Howitz P, Howitz J, Gjerris F. A variant of the Klippel–Trénaunay–Weber syndrome with temporal lobe astrocytoma. Acta Paediatr Scand 1979;68:119–121.
5. Spallone A, Tcherekaywv VA. Simultaneous occurrence of aneurysm and multiple meningioma in Klippel–Trénaunay patients: Case report. Surg Neurol 1996;45:241–244.
6. Bruart J, Parmentier R, Vanderhoeft P, Remacle P. A case of primary hemangiopericytoma associated with Klippel–Trénaunay–Parker–Weber syndrome. J Fr Med Chir Thorac 1971;25:145–155.
7. Weiss SW, Lasota J, Miettinen MM. Angiosarcoma of soft tissue. In: Fletcher CDM, Unni KK, Mertens F, editors. Pathology and genetics of tumours of soft tissue and bone. WHO Classification of Tumours. Lyon: IARC Press; 2002. pp 175–177.
8. Lezama-del Valle P, Gerald WL, Tsai J, Meyers P, La Quaglia MP. Malignant vascular tumors in young patients. Cancer 1998;83:1634–1639.
9. Oettler WJ, Ditter DD, Kruse HJ, Schellong SM. A huge malignant degenerating neurofibroma of the lower leg coexistent with type 1 Recklinghausen disease. Dtsch Med Wochenschr 2004;129:364–367.
10. Mentzel T, Katenkamp D. Intraneural angiosarcoma and angiosarcoma arising in benign and malignant peripheral nerve sheath tumours: Clinicopathological and immunohistochemical analysis of four cases. Histopathology 1999;35:114–120.
11. Rossi S, Fletcher CD. Angiosarcoma arising in hemangioma/vascular malformation of four cases and review of the literature. Am J Surg Pathol 2002;26:1319–1312.

